# Enhanced Synthesis of Foreign Nuclear Protein Stimulates Viral Reproduction via the Induction of *γ-Thionin* Expression

**DOI:** 10.3390/plants11121530

**Published:** 2022-06-07

**Authors:** Ekaterina V. Sheshukova, Natalia M. Ershova, Fedor A. Lipskerov, Tatiana V. Komarova

**Affiliations:** 1 Vavilov Institute of General Genetics Russian Academy of Sciences, 119991 Moscow, Russia; sheshukova@vigg.ru (E.V.S.); ershova@vigg.ru (N.M.E.); fedor@lipskerov.ru (F.A.L.); 2 Chemistry Department, Lomonosov Moscow State University, 119991 Moscow, Russia; 3 Belozersky Institute of Physico-Chemical Biology, Lomonosov Moscow State University, 119991 Moscow, Russia

**Keywords:** viral vector, defensin, γ-thionin, nuclear localization signal, nucleomodulin

## Abstract

Plants are a promising platform for recombinant protein production. Here we propose a novel approach to increase the level of viral vector-mediated recombinant protein synthesis. This approach is based on the hypothesis that antiviral protection is weakened during the antibacterial cellular response. We suggested that introduced to the cell foreign nuclear localized proteins, including effectors such as bacterial nucleomodulins, can interfere with the import of cellular nuclear proteins and launch antibacterial defense reactions, creating favorable conditions for cytoplasmic virus reproduction. Here, we performed synthesis of an artificial nuclear protein—red fluorescent protein (mRFP) fused with a nuclear localization sequence (NLS)—in plant cells as a mimetic of a bacterial effector. Superproduction of mRFP:NLS induced *Nicotiana benthamiana γ-thionin* (*NbγThio*) mRNA accumulation. Both NLS-containing protein synthesis and increased *NbγThio* expression stimulated reproduction of the viral vector based on the genome of crucifer-infecting tobacco mosaic virus (crTMV) in *N. benthamiana* leaves. We isolated the *NbγThio* gene promoter (Pr^γThio^) and showed that Pr^γThio^ activity sharply increased in response to massive synthesis of GFP fused with NLS. We conclude that NLS-induced Pr^γThio^ activation and increased accumulation of *Nbγthio* mRNA led to the stimulation of GFP expression from crTMV: GFP vector in the transient expression system.

## 1. Introduction

The development of methods for testing plant expression of foreign DNA enhanced modern understanding of plant cell functioning and stress responses [1]. Methods using isolated protoplasts [2], direct delivery of DNA to intact cells using particle bombardment [3,4], and methods for creating transgenic plants [5] facilitated the development of plant molecular biology. The agrobacterium-mediated transient plant transformation has become widely used for solving both fundamental and applied problems [6,7]. The design of the most widely used transient expression system consists of agroinfiltration of *Nicotiana benthamiana* leaves with an *Agrobacterium tumefaciens* cell suspension, followed by transfer of the gene (s) of interest to plant cells. Growing research demonstrates that this approach could be applied to other plant species [8,9]. Agrobacterial transformation affects the plant cell nucleus and gene expression pattern in the area of agroinfiltration, causing the cell to produce the protein of interest. Agrobacterium-mediated DNA delivery is highly efficient, transforming nearly all cells of the infiltrated leaf area and ensuring massive and synchronized production of the target protein [10,11,12]. *N. benthamiana* is used for biotechnological purposes, including recombinant protein production (for review see [13,14]). One approach that delivers high yield of the target protein is based on the use of viral vector-mediated expression [11,15,16,17,18]. Due to its ability to replicate, the viral vector is reproduced in the transformed cell with a greater number of copies compared to non-replicating expression vectors. However, plants have evolved numerous defense mechanisms against bacterial and viral invasion. For example, any foreign RNA, in particular viral RNA, is a target for cellular defense systems, including virus-induced gene silencing [19,20]. The use of silencing suppressors—viral proteins that interfere with the cellular silencing system—is a common tool in “green” biotechnology [21] p19 of *Tombusvirus*, p14 of *Auruvirus*, and HcPro of *Potyvurus* are just a few examples of silencing suppressors [22]. Agroinfiltration allows simultaneous delivery of several DNA fragments into one cell by mixing two or more bacterial cultures containing plasmids, making co-expression of the target gene with the silencing suppressor-encoding gene possible. To increase yield of the recombinant protein produced in the plant, additional approaches were developed to stimulate viral vector reproduction and system productivity: intron insertion into the viral vector-encoding plasmid and elimination of the putative cryptic introns [10], co-expression with short noncoding RNAs [23], plant sensibilization by gaseous methanol treatment [24], and incubation of the infiltrated plant in the dark [9]. Improvement of the plant transformation step also looks promising, as altering conditions during bacterial growth, the infiltration buffer, and plant incubation temperature are aimed to more efficient delivery of DNA to the plant cells [9]. Despite a variety of existing methods to increase the yield of target proteins in plant expression system there is a demand for further optimization and improvement of this production platform.

Here we propose a novel tool for stimulation of viral vector-mediated synthesis of the target protein. Our approach is based on the natural plant cell defense reactions in response to bacterial infection and in particular, nucleomodulins, the effectors that reprogram the nucleus of the host cell [25]. Many of the plant bacterial pathogens’ nucleomodulins enter the host cell nucleus via a nuclear-localization signal-mediated mechanism [26]. We hypothesize that introduced foreign nuclear localized proteins, including bacterial nucleomodulins, can interfere with the import of cellular nuclear proteins and launch antibacterial defense response, creating favorable conditions for cytoplasmic virus reproduction. Based on this suggestion we developed a novel approach to increase the level of viral vector-mediated recombinant protein synthesis by inducing NLS-containing proteins production, triggering antibacterial immunity and weakening antiviral protection.

## 2. Results

### 2.1. Massive Synthesis of a Model NLS-Containing Protein Stimulates Reproduction of a crTMV-Based Viral Vector

**Figure 1:**
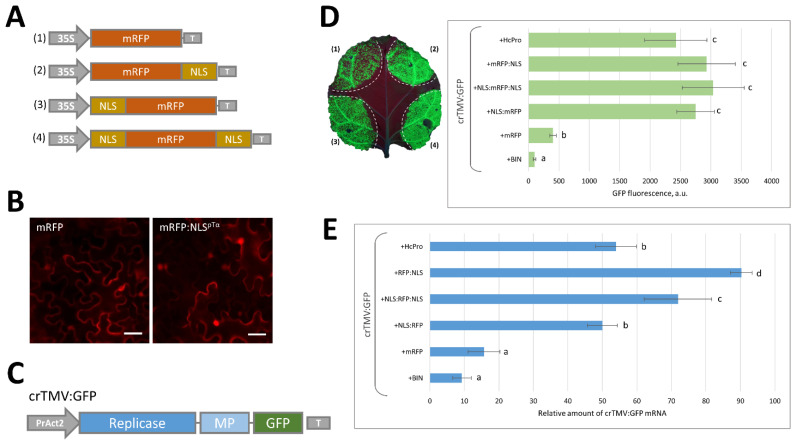
A foreign protein containing a nuclear localization signal (NLS) stimulates viral vector reproduction. (**A**) Schematic representation of the constructs encoding mRFP (**1**) or mRFP fused with a human prothymosin α NLS (**2**–**4**), a 35S RNA promoter (35S), and a terminator of transcription (T), where 35S and T were from the cauliflower mosaic virus; (**B**) fluorescent images of epidermal cells of *N. benthamiana* leaves agroinfiltrated with 35S-mRFP (**left**) or 35S-mRFP:NLS^pTα^ (**right**), scale bar = 20 μm; (**C**) schematic representation of the crTMV:GFP viral vector, from left to right is as follows: promoter of the *Arabidopsis thaliana ACT2* gene (PrAct2), genes encoding replicase and the movement protein (MP) of the crucifer-infecting tobamovirus (crTMV), and T; (**D**) GFP accumulation in *N. benthamiana* leaves 4 days after agroinfiltration with crTMV:GFP and an mRFP variant or silencing suppressor from potato virus A (HcPro):GFP fluorescence under UV light (**left**) and in plant extracts (**right**), a.u.—arbitrary units; (**E**) GFP mRNA accumulation in leaves 4 days after infiltration with crTMV:GFP and mRFP variants or HcPro and quantified by qRT-PCR. Histograms in (**D**,**E**) represent mean values with standard errors indicated. Bars with different letters indicate statistically significant differences at *p* < 0.01 (Students *t*-test).

We selected a bipartite nuclear localization signal (ESATGKRAAEDDEDDDVDTKKQKTDEDD) from human prothymosin α (pTα) (NLS^pTα^) [27] to obtain an artificial model NLS-containing reporter protein based on a red fluorescent protein (mRFP) sequence. We created a set of genetic constructs encoding mRFP/NLS fusion proteins in which an NLS was at the N-terminus, C-terminus, or both termini (Figure 1A). Using agrobacterium-mediated delivery of the genetic material for the transient expression, NLS^pTα^ effectively targeted mRFP to the nucleus (Figure 1B). We used a GFP-expressing crTMV-based vector crTMV:GFP to assess the effect of the synthesis of the large amount of NLS-containing proteins on viral reproduction (Figure 1C) [28]. *N. benthamiana* leaves were co-infiltrated with agrobacteria bearing the crTMV:GFP-encoding plasmid and one of the mRFP/NLS-encoding plasmids. GFP expression was visualized 5 days after inoculation (Figure 1D). All mRFP/NLS variants stimulated GFP accumulation, as shown by the GFP fluorescence measurement in the plant extracts (Figure 1D). All tested NLS-containing proteins had a similar effect on viral vector-mediated GFP production and stimulated GFP accumulation, which was at least five times greater compared to the variant without an NLS (mRFP). We used an HcPro silencing suppressor from potato virus A as a confirmed and conventional instrument for the stimulation of recombinant protein accumulation in *N. benthamiana* [29] for comparison.

The amount of synthesized GFP reflected the efficiency of crTMV:GFP reproduction, as confirmed by the assessment of viral RNA levels using quantitative real-time PCRs (Figure 1E).

### 2.2. NbγThio Expression Is Activated in Response to a Model NLS-Containing Protein

**Figure 2:**
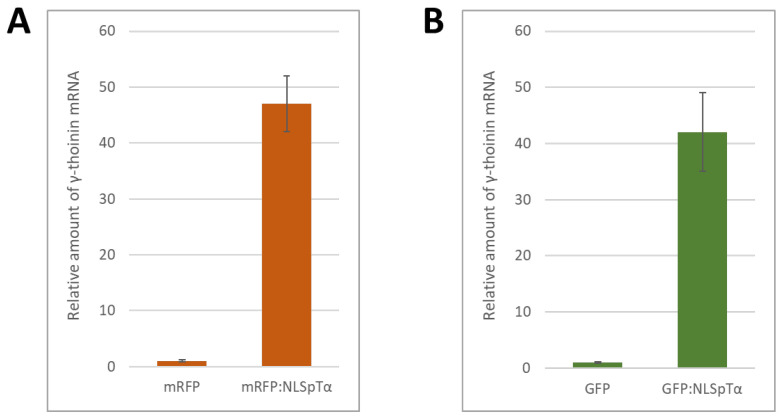
*N. benthamiana NbγThio* mRNA accumulation induced in response to the increased production of NLS-containing reporter proteins. The relative amount of *NbγThio* mRNA in leaves 3 days after agroinfiltration with 35S-based constructs encoding (**A**) mRFP or mRFP:NLS^pTα^ and (**B**) GFP or GFP:NLS^pTα^ as quantified by qRT-PCR.

We suggested that superproduction of a foreign nuclear protein was recognized by the cell as a signal for the induction of bacterial pathogenesis-related gene expression since NLS-containing proteins could be perceived by the plant cell as bacterial nucleomodulins, i.e., protein factors that are delivered to the nucleus to interfere with its functioning [30,31]. To identify the specific mechanism underlying the response to NLS-containing foreign proteins, we performed a suppressive subtractive hybridization and compared the mRNA profiles of leaves expressing 35S-mRFP versus 35S-mRFP:NLS^pTα^. From 96 analyzed clones 27 were upregulated in mRFP:NLS-expressing tissues (Table S1). Their sequences could be divided into three main groups: most of them (10) corresponded to unknown *N. benthamiana* proteins, 6 represented genes encoding different proteinase inhibitors, and 9 clones aligned with the same γ-thionin-like protein mRNA. Based on the sequence of these clones, we identified the corresponding *N. benthamiana* gene and designated it *NbγThio* (Gene Bank Acc. ON661791). To verify the subtractive hybridization data on γ-thionin expression we performed qRT-PCRs and confirmed that mRFP:NLS^pTα^ synthesis in the leaves drastically stimulated the accumulation of *NbγThio* mRNA (Figure 2A).

To exclude the effect of the reporter protein, we checked the *NbγThio* response to the nucleus-localized GFP:NLS^pTα^ (Figure S1) and assessed the level of *NbγThio* mRNA in leaves 3 days after agroinfiltration with 35S-GFP:NLS^pTα^. *NbγThio* expression increased comparably in response to both mRFP:NLS^pTα^ and GFP:NLS^pTα^ (Figure 2B).

Plant γ-thionins, or defensins, are relatively small proteins, belonging to the pathogenesis related (PR) protein PR-13 family and possess antifungal or antibacterial activity [32]. NbγThio has the characteristic features of γ-thionins [33,34]: an apoplast-targeting secretory signal peptide with eight cysteine residues in the mature protein that can form disulfide bonds and an amphipathic α-helix (Figure S2).

### 2.3. The NbγThio Promoter (Pr^γThio^) Is Sensitive to the Intensive Accumulation of the Model Nuclear Protein

**Figure 3:**
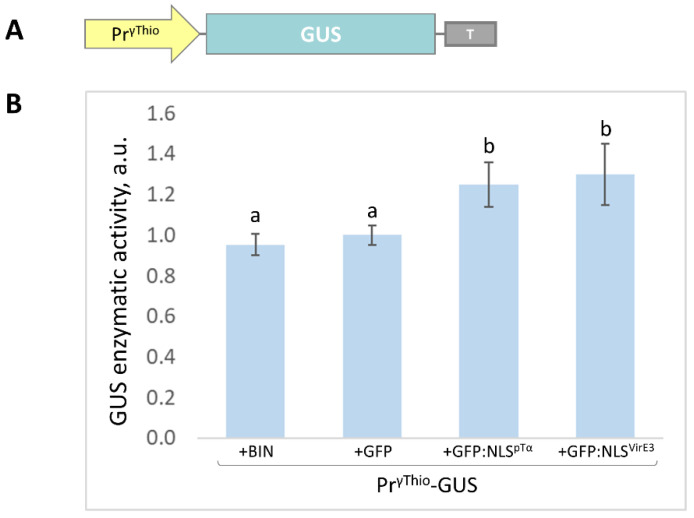
Artificial nuclear proteins (GFP:NLS^pTα^ or GFP:NLS^VirE3^) stimulated *NbγThio* promoter (Pr^γThio^)-directed GUS synthesis (Pr^γThio^-GUS). (**A**) Schematic representation of the Pr^γThio^-based vector encoding GUS; (**B**) comparative analysis of GUS activity in arbitrary units (a.u.) in leaves 3 days after co-agroinfiltration with Pr^γThio^-GUS and empty vector (+BIN) or 35S-based vectors encoding GFP, GFP:NLS^pTα^, and GFP:NLS^VirE3^. The fluorescence detected for the combination of Pr^γThio^-GUS and 35S-GFP was taken as 1. Standard error bars are indicated. Bars with different letters indicate statistically significant differences at *p* < 0.05 (Students *t*-test).

Using the “chromosome walking” approach [35], we isolated the 1142-nucleotide sequence upstream of the *NbγThio* gene, which we designated as the *NbγThio* promoter (Pr^γThio^). We then created plant expression vectors containing a reporter gene encoding *Escherichia coli* β-glucuronidase (GUS) under the control of Pr^γThio^ (Figure 3A). We used GFP fused with the NLS^pTα^ or *A. tumefaciens* virulence protein E3 [36] NLS (KRLRVDNPKELTREHGRLRKTKT) to model the entrance of bacterial NLS-containing nucleomodulins. NLS^VirE3^ effectively targeted GFP to the nucleus (Figure S1). According to our hypothesis on the stimulatory role of the foreign NLS-containing protein for NbγThio, the co-expression of Pr^γThio^-GUS with 35S-GFP:NLS^pTα^ or 35S-GFP:NLS^VirE3^ should result in increased GUS accumulation. We extracted GUS from agroinfiltrated leaves at 3 days post infiltration (dpi) and assessed its enzymatic activity, which reflected Pr^γThio^-GUS expression. Both GFP:NLS^pTα^ and GFP:NLS^VirE3^ synthesis activated Pr^γThio^ and stimulated GUS accumulation by approximately 20% (Figure 3B), indicating that Pr^γThio^ was sensitive to the foreign nuclear proteins. To check if this effect was specific to Pr^γThio^ but not to the constitutive promoter we performed co-agroinfiltration of 35S-GUS with 35S-GFP, 35S-GFP:NLS^pTα^, or 35S-GFP:NLS^VirE3^. We demonstrated that GUS production mediated by the 35S promoter was not stimulated by NLS-containing GFP and even decreased in response to it (Figure S3).

We concluded that *NbγThio* expression was stimulated at the level of transcription in response to the synthesis of a large amount of the foreign NLS-containing protein.

### 2.4. Co-Expression of crTMV:GFP Vector with 35S-NbγThio Resulted in the Increased Accumulation of Viral RNA and Enhanced GFP Production

**Figure 4:**
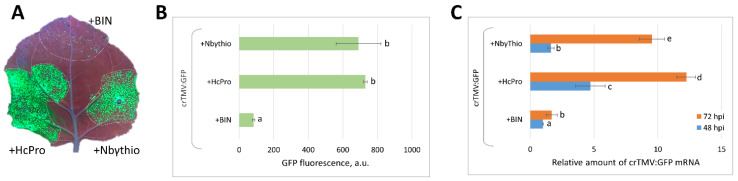
*N. benthamiana NbγThio* expression stimulated crTMV:GFP viral vector reproduction. (**A**) GFP fluorescence under UV light in leaves agroinfiltrated with crTMV:GFP and 35S-*NbγThio* (+NbγThio) or HcPro 5 dpi. Co-agroinfiltration with empty vector (+BIN) is regarded as a control; (**B**) GFP fluorescence in arbitrary units (a.u.) in plant extracts from agroinfiltrated leaves 5 dpi; (**C**) GFP mRNA accumulation in leaves 4 days after co-agroinfiltration with crTMV:GFP and 35S-*NbγThio* or BIN or HcPro as quantified by qRT-PCR. Histograms in (**B**,**C**) represent mean values with standard errors indicated. Bars with different letters indicate statistically significant differences at *p* < 0.05 (Students *t*-test).

We hypothesized that NLS-containing proteins could stimulate viral vector mRNA accumulation via the induction of NbγThio. To test this theory, we co-expressed crTMV:GFP with 35S-NbγThio and assessed the level of GFP accumulation (Figure 4A). Due to the GFP fluorescence measurement (Figure 4B) and the relative amount of viral RNA at 48 and 72 hpi (Figure 4C) it could be seen that 35S-NbγThio stimulated GFP expression from the viral vector.

We concluded that the super-expression of *NbγThio* promoted viral RNA accumulation.

## 3. Discussion

Over the past three decades, different plant-based systems have been developed and numerous valuable proteins have been produced in various plant species in proof-of-concept studies [37,38]. Among the diversity of plant platforms, the most common utilize *Nicotiana* species and transient expression in leaves [39]. Inconsistencies between expectations placed on so-called “molecular farming” and reality, in which this approach has met many hurdles, have led to some disillusionment. However, the plant platform remains attractive and there is a need to focus research on further development of potential instruments. With the use of a transient expression system involving viral vectors [15], it is possible to achieve high-yield, short-term recombinant protein production in a plant cell. Here we present instruments that allowed efficient target protein production comparable to that provided by the utilization of silencing suppressors. We demonstrated that rapid and massive accumulation of the NLS-containing foreign protein in the cell led to the creation of favorable conditions for cytoplasmic virus reproduction. We regarded this system as a model of bacterial invasion that affected both nucleocytoplasmic transport and the balance between antiviral and antibacterial cellular immunity. By comparison of the expression pattern in response to mRFP:NLS and mRFP accumulation, we revealed that one of the most abundant mRNA was the γ-thionin-like protein mRNA, the level of which was elevated during mRFP:NLS superproduction. Plant γ-thionins, or defensins, are relatively small proteins possessing antifungal or antibacterial activity [32]. These proteins are allocated to a separate group of *γ*-thionins mainly according to their secondary and tertiary structure rather than their amino acid sequence: they usually contain a signal peptide and several cysteine residues that form disulfide bonds that stabilize the αβ (CSαβ) motif [33,34,40]. The mechanism of γ-thionins’ antibacterial effect is still not completely understood, but their amphipathic helix and disulfide bonds are thought to play an important role [34]. In our model system, leaf mesophyll cells were likely perceive the artificial NLS-containing protein as a bacterial effector and hence as a signal of bacterial invasion. In response to the bacterial pathogen attack, the plant cell activated defense mechanisms, including the induction of *NbγThio* synthesis. We suggested that the increased level of *NbγThio* mRNA accumulation could be due to the activation of its transcription. By isolating Pr^γThio^ and using a reporter GUS-encoding gene, we demonstrated that this promoter was sensitive to accumulation of the foreign NLS-containing proteins. *GUS* expression increased in response to GFP:NLS^pTα^ and GFP:NLS^VirE3^ production, as these NLS-containing proteins mimicked bacterial effectors.

Our experiments also showed that the reverse side of the “explosive” synthesis of *NbγThio* mRNA was an increase in the sensitivity of the cell to the virus. By launching a variety of protective reactions in response to bacterial pathogens, a plant could “loosen” the antiviral line of defense. The mechanism of viral vector reproduction stimulation by NbγThio was not clear; it could be based on the effect of either NbγThio protein synthesis or *NbγThio* mRNA accumulation. Containing 318 nt, mature *NbγThio* mRNA is considered short. Massively synthesized short mRNA could affect nucleocytoplasmic transport competing with cellular mRNAs for the nuclear export factors, as this was demonstrated for short non-coding RNAs [23]. At the same time, the massive production of NLS-containing proteins could interfere with nucleocytoplasmic transport sequestering factors of the nuclear imports. Moreover, proper nucleocytoplasmic communication is a very important antiviral defense mechanism, for example, in virus-induced gene silencing. The impeded export of cellular mRNAs from the nucleus and the import of cytoplasmic proteins to the nucleus could lead to the creation of favorable conditions for cytoplasmic (+) RNA virus reproduction. We observed a similar situation in the plant’s response to gaseous methanol [24,41]. Gaseous methanol is emitted by wounded plant tissues and induces resistance to bacterial infection in the same plant and neighboring plants and stimulates intercellular transport. In addition, a side effect of this mobilization is increased sensitivity to viruses and favorable conditions for its intercellular spread and reproduction. We speculated that this was likely the case for a plant cell “choosing” the “lesser of two evils”, “preferring” a viral propagation and its persistent infection to cell death usually caused by pathogenic bacteria. Thus, the natural antibacterial immune response becomes an advantage for viral vector-mediated production of recombinant proteins and could be exploited as a basis for further optimization of the plant platform. Both NLS-containing proteins and NbγThio accumulation could be used to stimulate viral vector reproduction and increase yields of the recombinant protein in the transient expression system in *N. benthamiana* plants. An NLS-sensitive Pr^γThio^ could potentially be a basis for the creation of an inducible system for target protein production activated by bacterial nucleomodulins or other NLS-containing foreign proteins. Drawing on the mechanisms of maintaining a balance between plant resistance and susceptibility to pathogens we could develop new tools for green biotechnology and for valuable recombinant protein production in plant systems. The plant platform has unique advantages over well-established bacterial and mammalian expression platforms. It has great potential as the need for recombinant proteins for pharmacological and non-pharmacological purposes is increasing. The understanding of the principles underlying host–pathogen interactions generates a foundation for the development of novel approaches for the exploitation of plants as target protein production system.

## 4. Materials and Methods

### 4.1. Plant Growth Conditions

*N. benthamiana* plants were grown in soil in a controlled environment chamber in a 16 h/8 h day/night cycle.

### 4.2. Plasmid Constructs

For the 35S-mRFP constructs the mRFP sequence was amplified using primers “mRFP_SacI_d” and “mRFP_SalI_r”. A fragment was inserted into the pBIN19-based binary vector containing the *Cauliflower mosaic virus* (CaMV) 35S RNA promoter and the terminator of transcription via SacI/SalI sites. For the mRFP-NLS constructs the mRFP sequence without a stop codon and flanked with SacI and BamHI sites was obtained using primers “mRFP_SacI_d” and “mRFP_BamHI_r”. The plasmid 35S-mRFP-NLS^pTα^ was obtained via substitution of the GFP sequence with mRFP in the 35S-GFP-NLS^pTα^ plasmid [42], which was digested with SacI/BamHI restriction enzymes. To obtain 35S-NLS^pTα^-mRFP-NLS^pTα^ two PCR fragments were obtained: the NLS^pTα^ encoding sequence for N-terminal fusion was generated by annealing the primers “pTα_NLS_d” and “pTα _NLS_r”, resulting in a fragment with overhangs corresponding to the SacI and Acc65I “sticky” ends; the mRFP-encoding sequence was obtained using primers “mRFP_Acc65I_d” and “mRFP_BamHI_r” resulting in a fragment flanked with Acc65I and BamHI sites. Both fragments were inserted into the 35S-mRFP-NLS^pTα^ plasmid pretreated with SacI/BamHI restriction enzymes. The 35S-NLS^pTα^-mRFP constructs were made via substitution of the “mRFP-NLS^pTα^” sequence in the 35S-NLS^pTα^-mRFP-NLS^pTα^ plasmid with the mRFP fragment obtained by PCR with primers “mRFP_Acc65I_d” and “mRFP_SalI_r”.

For the 35S-GFP-NLS^VirE3^ constructs the NLS^VirE3^ encoding sequence was generated by annealing the primers “VirE3_NLS_d” and “VirE3_NLS_r”, resulting in a fragment with overhangs corresponding to BamHI and SalI “sticky” ends. That fragment was inserted into 35S-GFP-NLS^pTα^ via BamHI/SalI sites to substitute the NLS^pTα^ sequence.

The Pr^γThio^ fragment with HindIII and NcoI sites flanking it at the 5′- and the 3′-ends, respectively, was obtained as a product of the PCR with primers “Pr^γThio^ (HindIII+)” and “Pr^γThio^ (NcoI−)”. Then Pr^γThio^ was inserted into the 35S-GUS [42] plasmid using the HindIII and NcoI sites substituting the 35S-promoter sequence into it, resulting in the Pr^γThio^-GUS plasmid.

Oligonucleotide sequences are listed in Table S2.

### 4.3. Isolation of the γ-Thionin Promoter Region (Pr^γThio^)

Genomic DNA was isolated from plant tissues using the ZR Plant/Seed DNA MiniPrep™ kit (Zymo Research, Irvine, CA, USA). A GenomeWalker™ Universal Kit (Clontech, TaKaRa, Shiga, Japan) was used for two rounds of “genome walking” according to the manufacturer’s instructions. To identify the γ-thionin promoter region, the first round of walking was performed using the following oligonucleotides: PrT_Rev1, PrT_Rev2, and PrT_Rev3; the second round of walking was performed using the oligonucleotides PrT_Rev10, PrT_Rev11, and PrT_Rev12 (Table S2). The promoter region fragment was amplified using primers Pr_Rev2 and PrT_prom_Dir2 and subsequently cloned into the pAL-TA plasmid (Evrogen, Moscow, Russia). Pr^γThio^ EMBL Acc. ERA1901858.

### 4.4. Construction of SSH cDNA Libraries

Suppressive subtractive hybridization (SSH) was performed as described earlier [24]. Briefly, total RNA was isolated from the *N. benthamiana*-agroinjected leaves using TriReagent (MRC, Cincinnati, OH, USA). Amplified double-stranded cDNA was prepared using a SMART approach [43]. SMART-amplified cDNA samples were further digested by *Rsa* I endonuclease. Subtractive hybridization was performed in both directions (mRFP vs. mRFP:NLS^pTα^ and mRFP:NLS^pTα^ vs. mRFP). To eliminate background from SSH-generated libraries, the mirror orientation selection (MOS) method for both SSH subtracted libraries was exploited. Two subtracted cDNA samples, enriched with differentially expressed sequences obtained by MOS PCR, were used for construction of the library. At the next step the differential screening of the subtracted libraries was performed as described earlier [24].

### 4.5. Agroinfiltration Experiments

The *A. tumefaciens* strain GV3101 was transformed with individual binary constructs and grown at 28 °C in an LB medium supplemented with 50 mg/L rifampicin, 25 mg/L gentamycin, and 50 mg/L carbenicillin/kanamycin. The *Agrobacterium* overnight culture was diluted in 10 mM MES (pH 5.5) buffer supplemented with 10 mM MgSO_4_ and adjusted to a final OD_600_ of 0.1. Agroinfiltration was performed on almost fully expanded *N. benthamiana* leaves that were still attached to the intact plant. A bacterial suspension was infiltrated into the leaf tissue using a 2 mL syringe, after which the plants were grown under greenhouse conditions at 24 °C with a 16 h/8 h light/dark photoperiod.

### 4.6. GFP and mRFP Imaging

GFP and mRFP fluorescence in the inoculated leaves was monitored by illumination with a handheld UV source (DESAGA, Wiesloch, Germany). At higher magnifications, GFP fluorescence was detected using an LSM510 confocal laser scanning microscope (Zeiss, Jena, Germany). The excitation wavelengths for GFP and mRFP were 488 nm and 555 nm, respectively. The detection window was 500–550 nm for GFP and 590–650 nm for mRFP. Unless otherwise indicated, the lower epidermal cells of injected leaves were observed at 72 hpi.

### 4.7. GFP Fluorescence Measurement

Fifty milligrams of leaf tissue from infiltrated areas was ground in 1.5 mL tubes in 200 μL of GFP extraction buffer (150 mM NaCl, 10 mM Tris-HCl, pH 8.0). Then, the samples were centrifuged at 16,000× *g* for 10 min, and 1 mL of GFP extraction buffer was added to the supernatant. The fluorescence was measured using a Turner Quantech fluorimeter (Barnstead International, USA) with the following set of filters: NB390 (narrowband) excitation filter and an NB520 emission filter.

### 4.8. GUS Activity Measurement

GUS enzymatic activity in plant extracts was estimated with the substrate 4-methylumbelliferyl-β-D-glucuronide (MUG). The fluorescence of the MUG cleavage product was analyzed with a Turner Quantech fluorimeter (Barnstead International, Dubuque, IA, USA) using an NB455 (narrow band) excitation filter and an NB520 emission filter. All measurements were performed according to the previously described standard protocol [44] and measured in relative light units. GUS activity was normalized to the protein concentration estimated using a Bio-Rad protein assay kit. The mean values (with SE bars) for three independent experiments with three to ten biological repeats were shown.

### 4.9. Quantitative Real-Time PCR (qRT-PCR) Analysis of Transcript Concentrations

Total RNA was extracted from plant tissues using TriReagent (MRC, USA) according to the manufacturer’s instructions. The synthesis of the first strand, followed by real-time qPCR, was performed as described in [24]. Briefly, 0.2 µg of random hexamers and 0.5 µg of oligo-dT primer were added to 2 µg of total RNA to obtain cDNA through reverse transcription, performed using Superscript II reverse transcriptase (Invitrogen, Waltham, MA, USA), according to the manufacturer’s protocol. Real-time quantitative PCRs were carried out using the iCycler iQ real-time PCR detection system (Bio-Rad, Hercules, CA, USA). Reference genes were detected using the primers to 18S rRNA (18S_rRNA_dir ACGGCTACCACATCCAAG, 18S_rRNA_rev ACTCATTCCAATTACCAGACTC); protein phosphatase 2A-endcodinggene (NbPP2A_dir ATTGCTGCCTGTGGTTATTAC, NbPP2A_rev ATAGACTGAAGTGCTTGATTGG); elongation factor 1-alpha-encoding gene (EF1a_dir TCTTGAGGCTCTTGACCAG, EF1a_rev TTCCACACGACCAACAGG). Target genes were detected using the primers to NbγThio (Nb_thi_dir CCCTGGAATATGCCTTACC, Nb_thi_rev TCATCTTCTCATCAAACACAC) and GFP (GFP_dir GCAGAAGAACGGCATCAAG, GFP_rev GCTCAGGTAGTGGTTGTCG). Real-time qPCRs were performed using Eva Green master mix (Syntol, Russia) according to the manufacturer’s instructions. Each sample was run in triplicate, and a non-template control was added to each run. A minimum of five biological replicates were performed. The results of RT-qPCRs were evaluated using the Pfaffl algorithm [45].

## Data Availability

All data is provided in the manuscript.

## Supplementary Materials

The following supporting information can be downloaded at: https://www.mdpi.com/article/10.3390/plants11121530/s1, Figure S1: Fluorescent images of epidermal cells of *N. benthamiana* leaves expressing 35S-GFP, 35S-GFP:NLS^pTα^, or 35S-GFP:NLS^VirE3^; Figure S2: NbγThio secondary structure prediction by Phyre2 service [46]; Figure S3: Comparative analysis of GUS activity after co-agroinfiltration with 35S-GUS and 35S-based vectors encoding GFP, GFP:NLSpTα, and GFP:NLSVirE3; Table S1: Sequences of the clones identified in SSH as upregulated in response to mRFP:NLS; Table S2: Oligonucleotides used for cloning and “genome walking”.
