# Supplementary material for: Enhanced Synthesis of Foreign Nuclear Protein Stimulates Viral Reproduction via the Induction of γ-Thionin Expression
Source: Plants (Basel). 2022 Jun 7;11(12):1530. doi: 10.3390/plants11121530 (PMC9229031; doi:10.3390/plants11121530)
Supplement: Supplementary file 1 [file plants-11-01530-s001.zip › __FigS1-S3_TableS2.pdf]

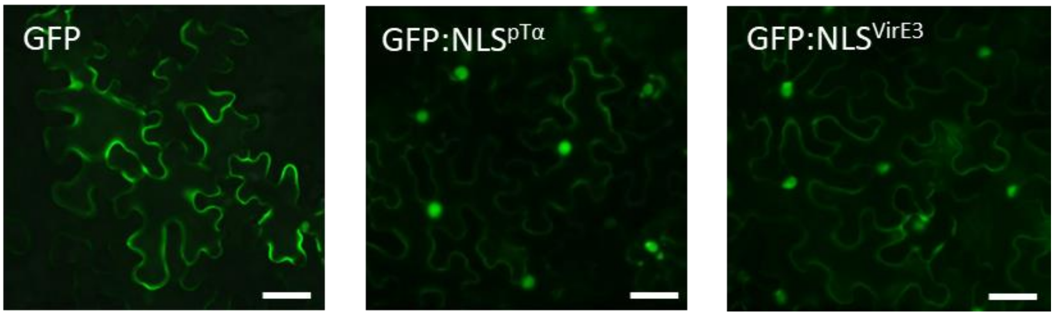

Figure S1. Fluorescent images of epidermal cells of *N. benthamiana* leaves agroinfiltrated with 35S-GFP (left), 35S-GFP:NLS$^{pT\alpha}$ (middle) or 35S-GFP:NLS^{VirE3} (right), scale bar = 20 µm

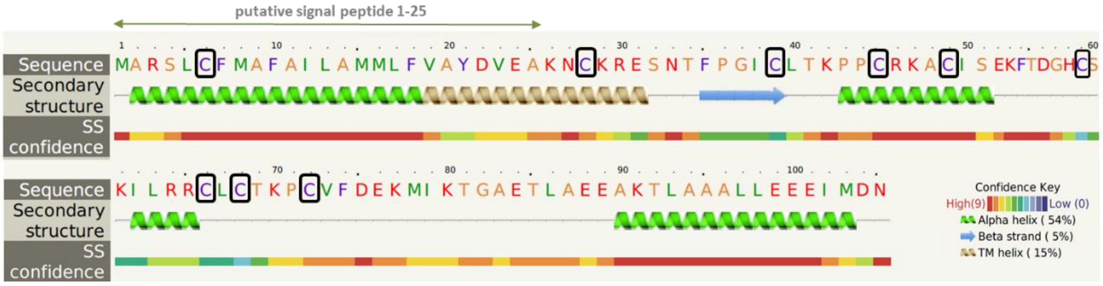

Figure S2. NbγThio secondary structure prediction by Phyre2 service [45] available at http://www.sbg.bio.ic.ac.uk/phyre2/html/page.cgi?id=index. Green helices represent α-helices, blue arrows indicate β-strands. The 'SS confidence' line indicates the confidence in the prediction, with red being high confidence and blue low confidence. The putative signal peptide was predicted by SignalP service (https://services.healthtech.dtu.dk/service.php?SignalP-5.0). Cysteines are outlined with black rectangles.

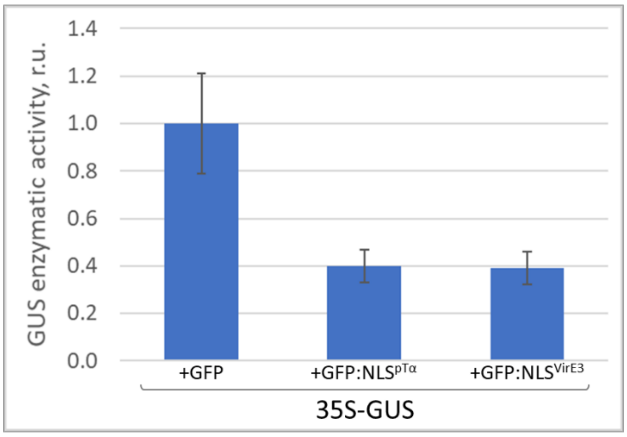

Figure S3. Comparative analysis of GUS activity in arbitrary units (a.u.) in leaves 3 days after co-agroinfiltration with 35S-GUS and 35S-based vectors encoding GFP, GFP:NLS$^{pT\alpha}$, and GFP:NLS^{VirE3}. The fluorescence detected for the combination of 35S-GUS and 35S-GFP is taken as 1.

Table S2. Oligonucleotides used for cloning and "genome walking"

Name	Nucleotide sequence
mRFP_SacI_d	GAGCTCATGGCCTCCTCCGAGGACGTC
mRFP_SalI_r	GTCGACTTAGGCGCCGGTGGAGTG
mRFP_BamHI_r	GGATCCGGCGCCGGTGGAGTGGC
mRFP_Acc65I_d	GGTACCGCCTCCTCCGAGGACGTC
pTα_NLS_d	GAGCTCGCAATGAGAATCTTTGAATTTG
pTα_NLS_r	GGTACCGTCATCCTCGTCGGTC
VirE3_NLS_d	GGATCCGGTTCTATAAGAACGAAACGGCTTCGAGTAGACAACCC AAAAGAATTAACGCGTG
VirE3_NLS_r	GTCGACTTACGTCTTGGTTTTGCGAAGTCTACCGTGCTCACGCGT TAATTCTTTTGGGTTG
$Pr^{\gamma Thio}$(HindIII+)	AAGCTTACCAGCACCTAAGCTCTCAT
$Pr^{\gamma Thio}$ (NcoI−)	ACAAGGAGCGAGCCATGGAGTTACTTTGAATGAGTAAAAAAGG
PrT prom Dir2	TTACCAGCACCTAAGCTCTCA
PrT_ Rev1	ATAGGCAACAAAGAGCATCATT
PrT_ Rev2	GAAGCACAAGGAGCGAGCCAT
PrT_ Rev3	AATAAGTAAAAGGAAATAATTATTGCA
PrT_ Rev10	TAACCTAGAACTCTAATACATGT
PrT_ Rev11	CATGACTTTAAATCGAGATTTCT
PrT_ Rev12	AACTGCACTACAATTCGTGAT
